# The search for relevant outcome measures for cost-utility analysis of systemic family interventions in adolescents with substance use disorder and delinquent behavior: a systematic literature review

**DOI:** 10.1186/s12955-017-0722-9

**Published:** 2017-09-19

**Authors:** S. Schawo, C. Bouwmans, E. van der Schee, V. Hendriks, W. Brouwer, L. Hakkaart

**Affiliations:** 1 Institute for Medical Technology Assessment & Institute of Health Policy & Management, Erasmus University Rotterdam, P.O. Box 1738, 3000 DR Rotterdam, The Netherlands; 2 Parnassia Addiction Research Centre (PARC), Brijder Addiction Treatment, Parnassia Bavo Group, Monsterseweg 83, 2553 RJ The Hague, The Netherlands; 3 Curium, Leiden University Medical Centre, Department of Child and Adolescent Psychiatry, Leiden University, Leiden, The Netherlands

**Keywords:** Economic evaluation, Instrument, Externalizing, Mental health, Youth

## Abstract

**Purpose:**

Systemic family interventions have shown to be effective in adolescents with substance use disorder and delinquent behavior. The interventions target interactions between the adolescent and involved systems (i.e. youth, family, peers, neighbors, school, work, and society). Next to effectiveness considerations, economic aspects have gained attention. However, conventional generic quality of life measures used in health economic evaluations may not be able to capture the broad effects of systemic interventions. This study aims to identify existing outcome measures, which capture the broad effects of systemic family interventions, and allow use in a health economic framework.

**Methods:**

We based our systematic review on clinical studies in the field. Our goal was to identify effectiveness studies of psychosocial interventions for adolescents with substance use disorder and delinquent behavior and to distill the instruments used in these studies to measure effects. Searched databases were PubMed, Education Resource Information Center (ERIC), Cochrane and Psychnet (PsycBOOKSc, PsycCRITIQUES, print). Identified instruments were ranked according to the number of systems covered (comprehensiveness). In addition, their use for health economic analyses was evaluated according to suitability characteristics such as brevity, accessibility, psychometric properties, etc.

**Results:**

One thousand three hundred seventy-eight articles were found and screened for eligibility. Eighty articles were selected, 8 instruments were identified covering 5 or more systems.

**Conclusions:**

The systematic review identified instruments from the clinical field suitable to evaluate systemic family interventions in a health economic framework. None of them had preference-weights available. Hence, a next step could be to attach preference-weights to one of the identified instruments to allow health economic evaluations of systemic family interventions.

## Background

Systemic family interventions are psychotherapeutic treatments, which are increasingly used to treat children and adolescents with mental disorders. These interventions are based on the idea that the behavior of a patient is the result of interactions between himself and the different ‘systems’ he is involved in (i.e. family, peers, school, etc.) and of the interactions between these systems [1–3]. Treatment is directed at improving the disturbing aspects within these interactions [3] and it actively involves the systemic context of the patient. Hence, potential effects are broad and may range from improvements in the interactions with parents, other family members, peers or neighbors, to improvements in educational achievements and work relations, reduction of criminal activity and substance use and reduction of problems with the juvenile justice system [2, 4–6]. Systemic family interventions have shown particularly effective in the treatment of adolescents with substance use disorders and delinquency [7–10]. Examples of these interventions are Multisystemic Therapy (MST), Functional Family Therapy (FFT), Multidimensional Family Therapy (MDFT) and Brief Strategic Family Therapy (BSFT) [7–10].

With the increasing use of systemic family interventions, the question of funding and reimbursement arises. In some countries, like the Netherlands or the United Kingdom, systemic family interventions are reimbursed from social health insurance schemes and, as such, are part of collectively financed health care. Hence, the interventions compete for limited funds with other health care expenditures and, on top of proving effective, need to demonstrate value for money. Common practice in the economic evaluation of medical interventions is the use of cost-utility analysis (CUA) [11, 12] measuring effects in terms of Quality-Adjusted Life-Years (QALYs). QALYs combine length and quality of life gained. Typically, quality of life is measured through preference-based, generic health outcome measures (such as the EQ-5D). These outcome measures typically concentrate on improvements in a number of health domains. A recent publication of our department [13] described the results of a CUA of MDFT versus Cognitive Behavioral Therapy (CBT) in which the effects were measured with the EQ-5D. Yet, in the field of mental health, doubts have been expressed [14, 15] on the use of these generic quality of life measures [16] as these tools might be too limited to cover all relevant treatment effects. Studies on the applicability of these measures in mental health have presented mixed results [14, 15]. Furthermore, there is increasing attention for the inclusion of spillover effects on caregivers and families in economic evaluations. Currently, these effects are not yet included [17, 18], though they may be particularly important in treatment of younger patients. Recently, the Second Panel of Cost-Effectiveness in Health and Medicine has recommended further research on quality of life effects on family members of patients [19].

Both aspects, the assessment of effects specific to mental health treatments and the inclusion of (partial) effects on third parties, seem of particular relevance to the economic evaluation of systemic interventions in delinquency and substance use in adolescents. As outcomes of systemic family interventions are broad and transcend health gains, conventional CUA outcome measures may be too limited and insufficiently connected to clinical practice. This may be one of the reasons why economic evaluations of systemic interventions are still scarce and overall of low quality [20]. Existing economic evaluations of these interventions vary in setting, design and in outcomes measured [20] hence limiting the comparability of results. Furthermore, few studies consider effects on others than the patient [1].

If the aim is to perform economic evaluations of systemic family interventions which account for all relevant effects, a disorder-specific multidimensional measure that captures all relevant systemic contexts would be desirable. Ideally, if such a measure had societal preference-weights attached to its dimensions and levels, it would deviate from the common CUA methodology yet enable CUA-like economic evaluations. In patients with substance use disorder (one of the patient groups treated with systemic family interventions), the need for such a single comprehensive outcome measure capturing the full benefits of treatments has been recognized before [21]. Deas and Thomas [22] and Hogue and Liddle [23] emphasized the necessity of assessing various outcomes beyond effects in the adolescent. In an illustrative pilot study, Jofre-Bonet and Sindelar [21] presented a first example of a preference-based measure for adult populations with substance abuse. However, that measure was not based on standard preference-elicitation techniques but the authors attached patient preference-weights to the eight main domains of the Addiction Severity Index (ASI) [24] by constructing a weight index.

**Fig. 1:**
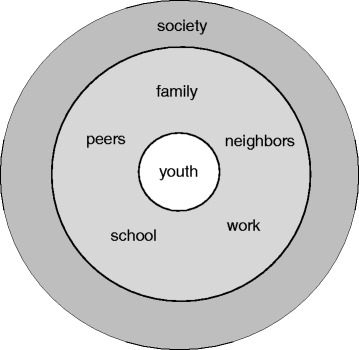
Systems involved in systemic family interventions for treatment of delinquency and substance-abuse in adolescents

In the current study, we take this line of research further by searching for a multidimensional outcome measure to evaluate systemic family interventions in the populations of adolescents with substance abuse disorder or problems of delinquency. Such a measure could facilitate CUAs of systemic family interventions and could either be based on existing effectiveness measures in this field or fully designed anew. In both cases, the use of an existing measure or the design of a new measure, relevant domains would need to be identified. Based on consultation of the literature on systemic family interventions [1, 25, 26] the domains relating to aspects of the individual patient, family, school (or work) and other community environments (e.g. peers, neighbors) were considered most relevant to the evaluation of the interventions. Figure 1 provides a graphical illustration of these domains, which indicate where potential effects may occur. The strength of the impact on the different systems may obviously differ, depending on the exact underlying problems and other contextual factors.

We perform a systematic literature review to investigate and appraise available instruments in the field of adolescent delinquency and substance use, which cover the relevant domains and which are already accepted and validated in the field. We assess which of these instruments might be most suited to serve as a basis for a preference-based measure in CUA, based on characteristics like comprehensiveness, brevity, accessibility, psychometric properties, etc. Advantage of using an existing instrument would be its being established, accepted and validated in the field and known by clinicians. It would then only be necessary to add preference-weights to the domains to account for differences in impact of each domain. In this way we aim to contribute to the development of adequate outcome measures to assess the economic value of systemic family interventions in the treatment of delinquency and substance use.

## Methods

We conducted a systematic literature review to identify instruments within the effectiveness and efficacy literature of mental health interventions for adolescents with substance use disorder and delinquency problems. We then assessed the suitability of these instruments for use of preference elicitation techniques. The assessment was based on several characteristics relevant to attain societal preference weights. These characteristics were among others the coverage of the systems displayed in Fig. 1 (i.e. youth, family, peers, school, work, society and neighbors), brevity, practicability of use, accessibility, psychometric properties and acceptance in the field. The review protocol was not registered. Yet, this study adhered to the PRISMA reporting guidelines [27].

### Criteria for inclusion

#### Types of participants

The target population of the systematic literature review consisted of adolescents between 12 and 18 years of age with symptoms of delinquency and/or substance use. Patients from specific sub-groups (e.g. homeless or runaway adolescents or adolescents with substance use disorder and comorbid depression) were excluded. As studies focusing on these subgroups evaluated specific outcomes, which were not necessarily relevant for the entire population of adolescents with substance use disorders and delinquent behavior, these studies were not considered relevant for the current study.

#### Types of interventions

We included studies on various mental health interventions for adolescents with substance use disorder or delinquency in a therapy/counseling setting in the systematic search to cover as many instruments as possible in the relevant target population. Individual interventions as well as systemic family interventions were included. Examples of such interventions are Cognitive Behavioral Therapy (CBT), Motivational Enhancement Therapy (MET), Multidimensional Family Therapy (MDFT), Multi Systemic Therapy (MST), Functional Family Therapy (FFT) and Ecologically Based Family Therapy (EBFT). Two types of interventions were excluded. First, interventions in mental health care that consisted of only pharmacotherapy were excluded since the focus of our study was specifically on the effect of psychosocial interventions. Second, mental health interventions for the prevention of criminal behavior or substance use disorder were excluded, as the symptoms within this group (i.e. high risk behavior or general behavioral problems) were not considered severe enough to fit the definition of the target population.

#### Types of outcome measures

Our objective was to identify a wide array of instruments used to measure the effect of mental health interventions for adolescents with substance use disorders and delinquent behavior. Hence, we included studies with all measures of effectiveness and treatment outcome as well as efficacy studies.

### Search methods for identification of studies

Databases were selected as to cover both interventions in the medical and in the educational field. The systematic literature review was performed in PubMed, Psychnet (PsycBOOKSc, PsycCRITIQUES, print), Cochrane and ERIC (Education Resource Information Center) to identify all effectiveness studies of mental health interventions for adolescent with substance use disorder or problems of delinquency. The databases were consulted between 5 March 2013 and 8 March 2013. Additional studies were identified based on reference list search. There were no restrictions on the type of publication. The language of publication was required to be English and publication date was 1990 or more recent. The search strategy used is displayed below.
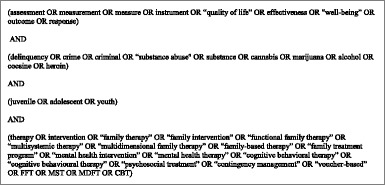


### Data analysis

#### Study selection

First, duplicates were removed. Then, the study selection was performed in two rounds. First, a selection based on title and abstract was performed, then selected articles were subject to a second screening based on full texts. Both rounds of selection were performed by two researchers independently and were each followed by a round of consensus. The eligibility criteria for the first selection based on title and abstract were the following.
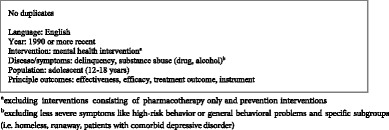


Subsequently, when abstracts or titles adhered to the above screening criteria, full texts were independently screened for inclusion based on the following (additional) criteria.
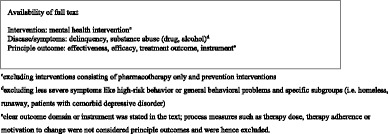


Furthermore, articles from reference lists of reviews were identified. For these, we performed a shortened screening and selection procedure. Titles of these articles were screened based on the following criteria: a) > =1990; b) peer-reviewed article; c) randomized control trial or effect/effectiveness/efficacy study/treatment outcome; d) adolescents; e) delinquency/offenders/substance-abuse; f) mental health intervention (no pharmacotherapy). If this selection resulted in inclusion, the abstract was screened and a final decision on inclusion or exclusion was made. Included articles were added to the database of identified articles for further data synthesis.

#### Data extraction

Data extraction was performed in MS Access with predefined fields. From all selected studies, general information, such as the title of the study, the name of the author, journal, etc., were recorded, as well as information on the sample size, the studied population and type of intervention (systemic, other [i.e. individual, group intervention], both).

In addition to this general information, instrument-specific information was extracted. This information consisted of instrument names (e.g. Child Behavior Checklist [CBCL]) and covered domains (e.g. family functioning, adolescent behavior, etc.). This information was recorded in order to identify the instruments currently used in the field and their coverage of the different systems relevant for the evaluation of systemic family interventions (Fig. 1).

#### Synthesis and evaluation of results

As a next step, domain names of the instruments were extracted from the identified articles and linked to the systems relevant for the evaluation of systemic family interventions (Fig. 1): youth, family, peers, school, work, society and neighbors. Domain names were verified with available resources such as guidelines, websites of the developer and other articles using the same instrument. After verification, the domains were translated into the systems mentioned in Fig. 1. For this purpose, domains related to the adolescents themselves, such as ‘substance use and abuse’, ‘physical health’ or ‘mental health’ were linked to the system ‘youth’ whereas domains such as ‘family relations’ were recoded into the system ‘family’, domains like ‘peer relations’, ‘social skills’ or ‘leisure/recreation’ were labeled as ‘peer’ system, domains like ‘educational status’ were labeled ‘school’ and ‘delinquency’ as ‘society’. Table 1 provides an example of the process of recoding for the Problem Oriented Screening Instrument for Teenagers (POSIT). Next, all instruments were classified based on the number of systems (presented in Fig. 1) covered and ranked from highest to lowest. Those covering five or more systems were considered most relevant for our purpose as those covered the majority of effects of systemic family interventions in adolescents with substance use disorder or problems of delinquency.

**Table 1:** Example of recoding of domains into systems

Problem Oriented Screening Instrument for Teenagers (POSIT)	
Domain	Corresponding system
Substance use and abuse	youth
Physical health	youth
Mental health	youth
Family relations	family
Peer relations	peers
Educational status	school
Vocational status	work
Social skills	peers
Leisure/recreation	peers,
Aggressive behavior/delinquency	society

In line with our aim to identify an instrument, which captures most of the systems relevant to the evaluation of systemic family interventions, those instruments covering more than five systems were evaluated in more detail. These were then appraised according to necessarily arbitrary characteristics of brevity, feasibility, practicability, accessibility, psychometric properties and acceptance in the field. These characteristics were set up as to identify one or more instruments suitable to attain societal preference-weights for an instrument by means of preference-elicitation techniques. Within preference-elicitation techniques, such as discrete choice experiments, the number of domains rarely exceeds ten [28, 29]. With higher numbers of domains, the decision task may become too complex and cognitively demanding for the respondent [28]. Hence, a suitable instrument should possess less than 10 domains. A second consideration was the practical use of the instrument itself in clients. An instrument, ideally suitable for self-completion, should put as little strain as possible on the respondent, without loss of important content. Hence, we set a limit to the maximum number of items of the instrument at 500 and a maximum completion time of 1 h, assuming that these would be reasonable amounts of items and time to ask from respondents. Another criterion was the accessibility of the instrument as to ascertain ease of use in future studies. Evaluation of this criterion included the price of use and availability of a (digital) version. Psychometric properties were considered to judge the suitability of the instrument for integration in health economic evaluations. Findings from existing publications on validity and reliability of the instruments were considered in this context. Finally, the frequency of use of the instrument was considered an indicator for the acceptance of the instrument in the clinical field. This was approximated by the number of times that an instrument was used in the studies identified in this review.

## Results

### Study selection

**Fig. 2:**
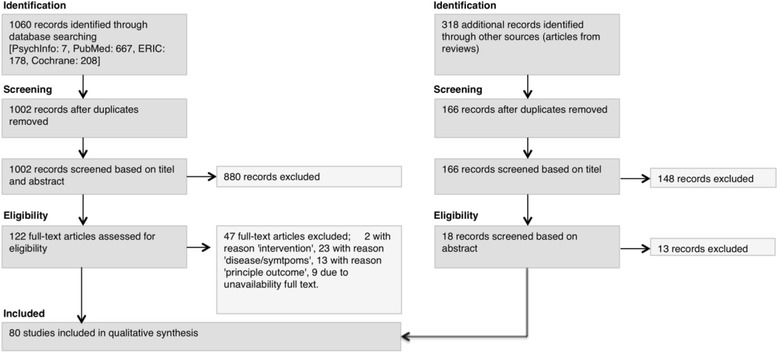
Phases of the systematic review adapted from Moher et al. [27]

The systematic search resulted in 1060 articles. After duplicates were removed 1002 articles remained. Screening based on abstracts resulted in the exclusion of 880 articles. Full text assessment of the remaining 122 articles resulted in the exclusion of two articles not matching the definition of the intervention, 23 articles not matching the disease or symptoms of the target population, 13 not matching the requirements for the principle outcome of the studies, and 9 due to unavailability of a full text version. Hence 75 articles were included. Furthermore, 318 underlying articles from reviews were screened. From these, 166 articles remained after duplications with the first search results were removed. The screening of these articles in a first round by title and in a second round by title and abstract resulted in the exclusion of 161 articles and inclusion of five additional publications (Fig. 2).

### Study results

A total of 80 articles were included in the synthesis. The aim was to identify clinical instruments in the field suitable for integration in a health-economic framework based on criteria of coverage of relevant systems, feasibility to perform preference-elicitation techniques, practicability of use, accessibility for future studies, psychometric properties and acceptance in the field. A summary of the identified reviews and clinical trials is provided in Tables 2 and 3 respectively. From the 80 selected articles we identified a total of 102 instruments, differing substantially in what these intended to measure and in whom. These instruments measured varying (combinations of) outcomes such as substance use, physical health, mental health, family relations, peer relations, school and work status and criminal history.

**Table 2:** List of identified reviews

ID	Authors	Year	Population
1	Armelius Bengt-Åke, Andreassen Tore Henning	2007	youth with antisocial behavior
2	Baldwin SA, Christian S, Berkeljon A, Shadish WR.	2011	adolescent delinquents and substance-abusers
3	Borduin CM.	1999	criminal and violent adolescents
4	Brown SA, D'Amico EJ.	2003	adolescent substance abusers
5	Cottrell D, Boston P.	2002	patients with conduct and attention deficit disorders, substance misuse, etc.
6	Curtis N, Ronan K, Borduin C M	2004	antisocial youths and youths with serious emotional disturbances
7	Deas D, Thomas SE.	2001	adolescents with substance use disorders
8	Deas D.	2007	adolescents with AOD disorders
9	Diamond G, Josephson A.	2005	adolescent substance use
10	Ferguson LM, Wormith JS.	2012	(adult and) young offenders
11	Henggeler SW, Sheidow AJ.	2012	conduct disorder and delinquency in adolescents
12	Henggeler SW, Sheidow AJ.	2003	conduct disorder and delinquency in adolescents
13	Hogue A, Liddle HA.	2009	adolescent substance abuse
14	Littell Julia H, Campbell Margo, Green Stacy, Toews Barbara	2005	(among others) delinquent youth
15	Randall J, Cunningham PB.	2003	violent substance-abusing and substance-dependent juvenile offenders
16	Tanner-Smith EE, Wilson SJ, Lipsey MW.	2013	adolescent substance use disorder
17	Tripodi SJ, Bender K, Litschge C, Vaughn MG.	2010	adolescent alcohol use
18	Waldron HB, Kaminer Y.	2004	adolescent substance use disorders
19	Waldron HB, Turner CW.	2008	adolescent substance abuse
20	Walker D F, McGovern S K, Poey E L, Otis K E	2004	adolescent sexual offenders
21	Woolfenden Susan, Williams Katrina J, Peat Jennifer	2001	adolescents with delinquency or conduct disorder

**Table 3:** Summary of identified clinical trials

ID	Authors	Year	Population	Age	Number	Effect measures
22	Arnold EM, Kirk RS, Roberts AC, Griffith DP, Meadows K, Julian J.	2003	incarcerated, sexually abused adolescent females	12–17	100	Multidimensional Adolescent Assessment Scale (MAAS)
23	Borduin CM, Mann BJ, Cone LT, Henggeler SW, Fucci BR, Blaske DM, Williams RA	1995	juvenile offenders	12–17	176	Symptom Checklist-90 (SCL-90-R);Revised Behavior Problem Checklist (RBPC); Family adaptability and cohesion evaluation scales-II (FACES-II); Nine-item unrevealed differences questionnaire-revised; Peer relations inventory (MPRI)
24	Borduin CM, Schaeffer CM, Heiblum N.	2009	juvenile sexual offenders	Mean 14	48	Global Severity Index (GSI) of the Brief Symptom Inventory (BSI); Revised Behavior Problem Checklist (RBPC); Family Adaptability and Cohesion Evaluation Scales II (FACES-II); 13-item Missouri Peer Relations Inventory (MPRI); Youth reports of the Self Report Delinquency Scale (SRD)
25	Brown SA, D'Amico EJ, McCarthy DM, Tapert SF.	2001	adolescents treated for alcohol and drug problems	14–18	166	Customary drinking and drug use record (CDDR); Hollingshead classification system; Health problems composite index
26	Burleson JA, Kaminer Y, Goldston DB, Haberek R	2005	Adolescent Alcohol Abuse or Alcohol Dependence Disorder	12–18 years; average 15.9	177	Suicidal Ideation Questionnaire (SIQ-JR); Teen Addiction Severity Index (T-ASI); Diagnostic interview scale for children (self-administered Voice-DISC)
27	Burleson JA, Kaminer Y.	2005	adolescents with substance use disorder	13–18	88	Situational confidence questionnaire (SCQ); Diagnostic interview schedule for children (DISC-C)
28	Cornelius JR, Douaihy A, Bukstein OG, Daley DC, Wood SD, Kelly TM, Salloum IM.	2011	adolescents with alcohol use disorder (AUD) and major depressive disorder (MDD)	15–20	75	Hamilton rating scale for depression (HAM-D-27); Beck Depression Index (BDI); Timeline follow-back method (TLFB)
29	D'Amico EJ, Ellickson PL, Wagner EF, Turrisi R, Fromme K, Ghosh-Dastidar B,Longshore DL, McCaffrey DF, Montgomery MJ, Schonlau M, Wright D.	2005	adolescents with alcohol and other drug problems (AOD)	13–19	289	Perceived stress scale; Revised way of coping checklist
30	Dembo R, Shemwell M, Guida J, Schmeidler J, Pacheco K, Seeberger W	1998	juvenile offenders	Mean 14	62	SCL-90-R
31	Dembo R, Wothke W, Livingston S, Schmeidler J	2002	juvenile offenders	11–18; mean 14.5	278	SCL-90-R
32	Dennis M, Godley S H, Diamond G, Tims F M, Babor T, Donaldson J, Liddle H, Titus J C, Kaminer Y, Webb C, Hamilton N, Funk R	2004	adolescent cannabis users	15–16	600	GAIN
33	Dennis M, Titus JC, Diamond G, Donaldson J, Godley SH, Tims FM, Webb C, KaminerY, Babor T, Roebuck MC, Godley MD, Hamilton N, Liddle H, Scott CK; C. Y. T.Steering Committee.	2002	cannabis dependence or abuse in adolescents	12–18	600	Drug Abuse Treatment Cost Analysis Program (DATCAP); Global Appraisal of Individual Needs (GAIN); Adolescent Reasons for Quitting (ARFQ); Family Environment Scale (FES); Friends, Family and Self (FFS); Adolescent Relapse Coping Questionnaire (ARCQ); SCID II personality questionnaire (SPQ); Dimensions of Temperament Revised (DOTS); Child Behavioral Checklist (CBCL)
34	Gil AG, Wagner EF, Tubman JG	2004	juvenile offenders	14–19	213	Timeline follow-back interview (TLFB);Problem recognition questionnaire (PRQ)
35	Glisson C, Schoenwald SK, Hemmelgarn A, Green P, Dukes D, Armstrong KS, Chapman JE	2010	delinquent youth	9–17	child behavior checklist (CBCL)	Child behavior checklist (CBCL)
36	Godley SH, Garner BR, Passetti LL, Funk RR, Dennis ML, Godley MD.	2010	adolescents with substance use disorder	12–18	320	GAIN Substance Problem Scale (SPS); DATCAP
37	Godley SH, Hedges K, Hunter B.	2011	adolescents with substance use	10–18	2141	GAIN
38	Hall JA, Smith DC, Easton SD, An H, Williams JK, Godley SH, Jang M	2008	youth in outpatient treatment for substance abuse	12–18	404	GAIN
39	Harold GT, Kerr DC, Van Ryzin M, Degarmo DS, Rhoades KA, Leve LD.	2013	adolescent girls in juvenile justice system	13–17	166	Brief symptom inventory (BSI)
40	Henderson CE, Dakof GA, Greenbaum PE, Liddle HA.	2010	adolescent drug abuse and delinquency	12–17	378	Personal Experience Inventory (PEI); Timeline follow-back method (TLFB); Diagnostic interview schedule for children, second edition (DISC-2); Family environment scale (FEI)
41	Henggeler SW, Halliday-Boykins CA, Cunningham PB, Randall J, Shapiro SB, Chapman JE.	2006	juvenile offenders meeting criteria for substance abuse or dependence	12–17	161	Form 90 based on TLFB; Self reported delinquency scale (SRD); Child behavior checklist (CBCL)
42	Henggeler SW, McCart MR, Cunningham PB, Chapman JE.	2012	youth substance abuse and criminal behavior	12–17	104	Form 90/TLFB; Self reported delinquency scale (SRD)
43	Henggeler SW, Melton GB, Brondino MJ, Scherer DG, Hanley JH	1997	violent and chronic juvenile offenders	11–17	155	Global severity index (GSI) of the BSI; Revised problem behavior checklist (RBPC); Self-report delinquency scale (SRD); Family adaptability and cohesion evaluation scales (FACES-III); Family assessment measure (FAM-III); Parent version monitoring index; Adolescent version monitoring index; 13-item Missouri peer relations inventory (MPRI); 14-item parent peer conformity inventory (PPCI)
44	Henggeler SW, Melton GB, Smith LA	1992	serious juvenile offenders	Mean 15,2	84	SRD (Self Report Delinquency Scale); FACES-III (The Family Adaptability and Cohesion Evaluation Scales); MPRI (Missouri Peer Relations Inventory); RBPC (Revised Behavior Problem Checklist); SCL-90 (Self Report Symptom Checklist); SCS-CBC
45	Henggeler SW, Pickrel SG, Brondino MJ.	1999	substance-abusing and -dependent delinquent adolescents	12–17	118	Personal experience inventory (PEI); Self-report delinquency scale (SRD)
46	Hogue A, Dauber S, Stambaugh LF, Cecero JJ, Liddle HA.	2006	substance-abusing adolescents	average 15.5	100	TLFB; CBCL; YSR
47	Hogue A, Henderson CE, Dauber S, Barajas PC, Fried A, Liddle HA.	2008	adolescent substance use and related behavior problems	13–17	136	Timeline follow-back (TLFB); Personal experience inventory (PEI); CBCL; YSR
48	Hunter SB, Ramchand R, Griffin BA, Suttorp MJ, McCaffrey D, Morral A.	2012	adolescent substance use	n/a	2751	GAIN; Emotional problem scale (EPS); Illegal activities scale (IAS); Past month substance problem scale (SPS-GAIN); Substance frequency scale (SFS-GAIN)
49	Kaminer Y, Burleson JA, Goldberger R	2002	adolescent substance-abusers	13–18	88	T-ASI; DISC-C; Structural clinical interview for the DSM (SCID-II); Revised dimensions of temperament survey (DOTES-R)
50	Kaminer Y, Burleson JA.	2008	adolescents with substance use disorder	13–18	88	T-ASI; DOTES-R
51	Keiley MK.	2007	incarcerated adolescents	13–18	73	Caregiver CBCL; Youth self-report (YSR); Coping inventory of stressful situations (CISS); Parental bonding instrument (PBI); Inventory of parent and peer attachment (IPPA)
52	Killeen TK, McRae-Clark AL, Waldrop AE, Upadhyaya H, Brady KT.	2012	adolescents with marijuana use disorders	12–18	31	Composite international diagnostic interview (CIDI); Teen Addiction Severity Index (T-ASI); Timeline Follow-back (TLFB); Marijuana craving questionnaire (MCQ-12); Barrett Impulsivity Scale (BIS-II-A)
53	Latimer WW, Winters KC, D'Zurilla T, Nichols M	2003	adolescent drug abusers	12–18	43	Diagnostic interview for children and adolescents (DICA-IV); Adolescent diagnostic interview revised (ADI-R)-multiaxal interview; Personal experience inventory (PEI); Family assessment measure (FAM); Social problem solving inventory (SPSI); Motivational learning questionnaire (MSLQ); Client personal history questionnaire (CPHHQ)
54	Letourneau EJ, Henggeler SW, Borduin CM, Schewe PA, McCart MR, Chapman JE, Saldana L	2009	juvenile sexual offenders	11–17	67	Adolescent sexual behavior inventory (ASBI); Self-report delinquency scale (SRD); Personal experience inventory (PEI); Child behavior checklist (CBCL)
55	Liddle HA, Dakof GA, Parker K, Diamond GS, Barrett K, Tejeda M	2001	clinically-referred marijuana- and alcohol-abusing adolescents	13–18	182	Adolescent grade point average (GPA); Acting out behaviors (AOB) scale; Global health pathology scale
56	Liddle HA, Rowe CL, Dakof GA, Henderson CE, Greenbaum PE	2009	young adolescent substance abusers	11–15	83	GAIN; TLFB; POSIT; SRD; National youth survey peer delinquency scale
57	Liddle HA, Rowe CL, Dakof GA, Ungaro RA, Henderson CE.	2004	adolescent substance abuse and behavioral problems	11–15	80	GAIN; Parent and adolescent interviews (CTRADA); Youth Self Report (YSR); Family Environment Scale (FES); National Youth Survey Peer Delinquency Scale; TLFB
58	Lott DC, Jencius S.	2009	adolescent substance abuse	12–18	264	(only urine specimen)
59	Marsden J, Stillwell G, Barlow H, Boys A, Taylor C, Hunt N, Farrell M	2006	young ecstasy and cocaine users	16–22	342	Maudsley addiction Profile (MAP); Severity of dependence scale (SDS)
60	Martin G, Copeland J.	2008	adolescent cannabis users	14–19	40	TLFB; Items from GAIN; Severity of dependence scale (SDS); Stage of change questionnaire
61	McCambridge J, Strang J.	2004	adolescent illegal drug use	16–20	200	Severity of Dependence Scale (SDS); Seven-point scale by Argyle; Drug Attitudes Scale (DAS); 12-item general health questionnaire (GHQ)
62	McGlynn AH, Hahn P, Hagan MP.	2012	juvenile offenders	12–18	518	HIT questionnaire
63	Moore SK, Marsch LA, Badger GJ, Solhkhah R, Hofstein Y.	2011	opoid-dependent adolescents	13–18	36	Youth Self Report (YSR)
64	Rigter H, Henderson CE, Pelc I, Tossmann P, Phan O, Hendriks V, Schaub M, RoweCL.	2012	adolescents with recent cannabis use disorder	13–18	450	Adolescent diagnostic interview-light (ADI-Light); TLFB
65	Robbins MS, Feaster DJ, Horigian VE, Rohrbaugh M, Shoham V, Bachrach K, Miller M,Burlew KA, Hodgkins C, Carrion I, Vandermark N, Schindler E, Werstlein R,Szapocznik J.	2011	adolescent drug abuse	Mean 15.5	480	TLFB; Diagnostic interview schedule for children (DISC); Parenting Practices Questionnaire; Family environment scale (FES)
66	Rohde P, Jorgensen JS, Seeley JR, Mace DE	2004	incarcerated youth	12–25	76	YSR; Life attitudes schedule-short form (LAS-SF); Self-esteem scale; 10-item UCLA loneliness scale; Subjective probability questionnaire; 4 items created and modeled after the social adjustment scale
67	Sawyer AM, Borduin CM	2011	serious and violent juvenile offenders	37,3	176	(no instruments, clinical records only)
68	Sealock MD, Gottfredson DC, Gallagher CA	1997	substance-abusing youthful offenders	n/a	460	Face valid alcohol (FVA) scale of SASSI; Face valid other drug (FVOD) scale of SASSI; Coping resources inventory (CRI); MEPS test
69	Sexton T, Turner CW	2010	adolescents adjudicated for crime and sentenced to probation	13–17	917	WAJCA-RA structured interview
70	Timmons-Mitchell J, Bender MB, Kishna MA, Mitchell CC	2006	juvenile justice involved youth	Mean 15.1	93	Child Adolescent Functional Assessment Scale (CAFAS)
71	Waldron HB, Kern-Jones S, Turner CW, Peterson TR, Ozechowski TJ.	2007	treatment resistant drug-abusing adolescents	14–20	72	BDI; State-anger subscale of the state-trait anger expression inventory (STAXI); State-anger anxiety inventory (STAI); TLFB; CBCL; YSR; Conflict and cohesion subscale of FES
72	Waldron HB, Slesnick N, Brody JL, Turner CW, Peterson TR.	2001	adolescent substance abuse	13–17	114	TLFB; POSIT; CBCL
73	Walker DD, Stephens R, Roffman R, Demarce J, Lozano B, Towe S, Berg B.	2011	adolescent cannabis users	14–19	311	Global Appraisal of Individual Needs (GAIN-I); Marijuana Problem Inventory (MPI) (adapted from RAPI)
74	Winters KC, Fahnhorst T, Botzet A, Lee S, Lalone B	2012	Alcohol and/or cannabis use disorder	12–18	315	PCS: 11-item self-report scale from the Personal Experience Inventory (PEI); Adolescent Diagnostic Interview (ADI); TLFB; Stages of Change Readiness and Treatment Eagerness Scale (SOCRATES); Problem solving inventory; Child version of the Alabama Parenting Questionnaire (APQ); Treatment Services Review (TSR)
75	Winters KC, Leitten W.	2007	drug-abusing adolescents	14–17	79	Adolescent Diagnostic Interview (ADI) - substance use disorder module; TLFB; Personal consequences scale (PCS) from PEI; Treatment Services Review (TSR)
76	Chamberlain P, Leve LD, Degarmo DS	2007	Girls with serious and chronic delinquency	13–17	81	Elliott General Delinquency Scale
77	Liddle, HA, Dakof, GA, Turner, RM, Henderson, CE, Greenbaum, PE	2008	Youth with drug abuse/dependence	Mean 15	224	DISC; PEI; TLFB
78	Robbins MS, Szapocznik J, Dillon FR, Turner CW, Mitrani VB, Feaster DJ	2008	Substance-abusing or dependent adolescents	12–17, mean 15.6	190	DISC; TLFB; ADAD; Therapist Adherence Checklist
79	Smith DC, Hall JA, Williams JK, An H, Gotman N.	2006	Adolescent substance abuse	Mean 15.8	98	SFS and SPS scales of GAIN
80	Winters KC, Stinchfield RD, Opland E, Weller C, Latimer WW.	2000	Adolescent substance abuse	12–18	245	Drug consumption items of PEI

#### Instrument suitability for evaluation of systemic family interventions

Table 4 displays the instruments ranked according to the number of systems covered.

**Table 4:** Ranking of instruments according to the number of systems covered

Name instrument	# systems covered	Systems						
		youth	family	peers	school	work	society	neighbors
POSIT	6	●	●	●	●	●	●	
CAFAS	6	●	●	●	●	●	●	
WAJCA-RA	6	●	●	●	●	●	●	
ADAD	6	●	●	●	●	●	●	
T-ASI	6	●	●	●	●	●	●	
CTRADA	5	●	●	●	●		●	
ADI	5	●	●	●	●		●	
GAIN	5	●	●		●	●	●	
PEI	4	●	●	●	●			
MAAS	4	●	●	●	●			
FES	4	●	●	●	●			
CPHHQ	3			●	●			●
FFS	3	●	●	●				
SCQ	3	●		●		●		
SRD	2	●					●	
SCL-90-R	2	●	●					
BSI	2	●	●					
Hollinghead classification system	2				●	●		
IPPA	2		●	●				
CRI	2	●	●					
MAP	2	●		●				

*Note.* • = system covered by instrument

The majority, 81 instruments, covered just one system such as the youth or the family system. These one-dimensional instruments were often used in a multi-method (i.e. a combination of self-report, parent-report, court records, urine-analysis, etc.) assessment battery of instruments. Fourteen instruments covered two, three or four systems. We identified eight instruments, which covered five or more systems and which therefore were considered potentially suitable for comprehensive evaluation of systemic family interventions.

Detailed information on these eight instruments was searched and is highlighted below. It has to be noted that available information per instrument (e.g. number of items, example questions, domain names, most recent versions of the instrument, type of administration, etc.) strongly differed.

The **Adolescent Drug Abuse Diagnosis (ADAD**) [30] is a multidimensional instrument to evaluate adolescent substance use [31] administered in a structured interview. It covers nine problem areas: medical, school, employment, social relations, family and background relations, psychological, legal, alcohol use, and drug use [32]. Example questions are “How would you rate your overall physical health?”, “How many days in the past 30 have you been absent (from school)?” and “How many months did you work fulltime in the past six months?”. A patient’s treatment need is assessed by the interviewer per problem area based on a 10-point rating scale with scores 0–1 (no real problem), 2–3 (slight problem, treatment probably not necessary), 4–5 (moderate problem, some treatment indication), 6–7 (considerable problem, treatment necessary), and 8–9 (extreme problem, treatment absolutely necessary) [32]. The instrument consists of 150 items and is based on the Addiction Severity Index (ASI) [24]. There is also a European version of the instrument, the European Adolescent Assessment Dialogue (EuroADAD). Its aim is to “describe, communicate and compare young clients over borders of countries and institutions.” [33].

The **Adolescent Diagnostic Interview (ADI)** [34] originated in the 1980’s as a project “to address measurement gaps in the alcohol-drug field” [35]. It is a tool to measure substance use disorders in adolescents “…organized around *DSM–III–R* criteria for psychoactive substance use disorders.” [34]. In the literature a version based on DSM-IV criteria is also mentioned [36]. The instrument is administered in a structural interview setting. Substance use of the adolescent is assessed based on two main sections with each two subsections: clinical (sociodemographics, psychosocial stressors, substance use frequency and duration, alcohol symptoms, cannabis symptoms, other substance symptoms and level functioning) and appendix (orientation and memory screen) [34]. Example items are “Which drugs have you used five or more times in your life?”, “How many times do you think that you have used (this drug/each drug) in the past 6 months?”, “Have you ever continuously felt like crying for several days in a row?” [36]. A computer-based version is available for self-assessment [34].

The **Child Adolescent Functional Assessment Scale (CAFAS)** “…assesses the degree of impairment in functioning in children and adolescents secondary to emotional, behavioral, or substance use problems” [37]. The instrument originally included seven scales, of which five evaluated the functioning of the youth and two scales assessed the environment of the youth [37]. The five youth scales were role performance, thinking, behavior towards self and others, moods/emotions, and substance use [37]. The two environment scales were basic needs and family/social support. The scales subsequently have been changed and expanded to 8 youth and 2 caregiver scales: school, home, community, behavior towards others, moods, self-harm, substance use, and thinking (youth) and material needs, and social support (caregiver) [38]. The different subscales include items of four severity levels (i.e. severe, moderate, mild, and minimal or no impairment) [37]. The assessor determines the level of problems of the patient per subscale. He first considers the items of the most severe level, checks whether these items apply and if not progresses towards the lesser symptom levels until an item of the current severity level applies to the patient [37]. Then scores of 30, 20, 10 and 0 are applied to severity levels severe, moderate, mild and minimal respectively such that an overall severity rating is generated. Overall ratings range from 0 to 240 with higher scores indicating higher severity [30].

The **Global Appraisal of Individual Needs (GAIN)** questionnaire [39] is a collection of related instruments that are gathered under the umbrella of GAIN using an identical format. The most recent version of the questionnaire has been adapted for use in adults as well as adolescents. The GAIN is an assessment measure, which can be used in several settings and populations such as inpatient, outpatient short- or long-term treatment evaluation, legal programs or school-based programs [40]. It assesses eight domains: background, substance use, physical health, risk behaviors, mental health, environment, legal, and vocational. Example items of the GAIN are “During the past 90 days, on how many days were you in foster care?”, “When was the last time, if ever, you used...any kind of alcohol?”, and “What was the most (drinks/joints/etc.) you had in one day?” [41].

The **Problem Oriented Screening Instrument for Teenagers (POSIT)** is a screening instrument for adolescents with substance use disorder, which was designed as a component of the Adolescent Assessment/Referral System (AARS) [42]. It “is designed to flag those functional areas, if any, where a problem MAY exist that requires further assessment and perhaps treatment.” [42]. The instrument addresses ten functional domains: substance use/abuse, physical health status, mental health status, family relations, peer relations, educational status, vocational status, social skills, leisure and recreation, and aggressive behavior and delinquency. The POSIT includes 139 items, which can be answered with yes or no [42]. Per domain, items can be grouped into three categories: general purpose items, general purpose age-related items, and red flag items [42]. Each affirmative response to a general purpose item counts as one point towards the total functional domain score [42]. The same holds for general purpose age-related items, but these are only relevant for specific age groups of respondents (below or above 16 years) [42]. Red flag items indicate the need for treatment once one of these items is answered positively [42]. Example items of the POSIT are “Do you get into trouble because you use drugs or alcohol at school?”, “Do your parents or guardians argue a lot?”, and “Have you ever been told you are hyperactive?” [42].

The **Teen Addiction Severity Index (T-ASI)** [43] is the adolescent version of the ASI [24]. The instrument assesses seven dimensions of functioning (i.e. alcohol and drug use, school status, employment-support status, family relationships, legal status, peer-social relationships, and psychiatric status) [43]. The T-ASI is intended for use in adolescents with substance use disorder aged between 12 and 19 years [43]. Example items of the T-ASI are “What chemicals have you used in the past month?”, “School days spent in detention or any other measures taken for disciplinary reasons last month. (Principal's or school counselor's office.)”, and “How long was your longest period of employment during the past year?” [44]. Responses are rated on a 5-point scale [43]. A revised version of the T-ASI, the T-ASI-2 has been developed in 2008. This concerns a version of the instrument, which is self-administered via computer or telephone and contains additional domains [45].

The **WAJCA-RA structured interview** is a risk assessment tool for juvenile offenders developed by the Washington State Institute for Public Policy in collaboration with the juvenile courts [46]. It was designed to identify risk and protective factors in the following domains: criminal history, school, use of free time, employment, relationships, family, alcohol and drugs, mental health, attitudes, social skills, progress on community supervision, progress while confined [46]. Example items of the WAJCA-RA are “Violence/anger: Reports of displaying a weapon, fighting, threatening people, violent outbursts, violent temper, fire starting, animal cruelty, destructiveness, volatility, intense reactions.”, “Runaways or times kicked out of home”, and “Number of weeks of longest period of employment” [46].

The **Parent and adolescent interview CTRADA** that was used by Liddle et al. [47] was not considered a common instrument but institution-specific interview as no references could be retrieved from neither literature nor the Internet. The instrument therefore could not be further considered or assessed.

#### Instrument suitability for use in CUA

Hence seven instruments remained for further consideration. The frequency of use of each of these instruments in the identified studies is presented in Table 5. Furthermore, Table 6 illustrates an evaluation of the instruments for suitability for use in CUA and use of preference elicitation techniques. When our feasibility characteristics were applied to the seven instruments, three instruments (POSIT, WAJCA, ADI) were excluded due to the number of domains exceeding ten, and one instrument (GAIN) was excluded due to reasons of practicability (i.e. number of items exceeding the maximum of 500 and completing time exceeding 1 h). It was noted that a short version of the GAIN (Global Appraisal of Individual Needs Short Screener, GAIN-SS) is available as well [48]. However, based on its goals of screening, use for clinical staff with limited experience or periodic measurement [48], this instrument is considered too restricted for the purpose of this study. The remaining three instruments (CAFAS, T-ASI and Euro-ADAD) were considered candidates for use in CUA. One instrument (CAFAS) was considered slightly less suitable due to reasons of accessibility (i.e. concerning a paid instrument as opposed to freely available online versions of other instruments). For the remaining two instruments (T-ASI and the Euro-ADAD) only limited information on psychometric properties could be obtained. It needs noting that the T-ASI and Euro-ADAD are related as they are both based on the ASI adult instrument [33, 43]. Psychometric properties of this ‘predecessor’ have been judged satisfactory [24, 49–52]. To our knowledge Euro-ADAD is more frequently used in Europe, whereas T-ASI is more commonly used in the United States.

**Table 5:** Frequency of instrument use

Instrument name	# of papers which used this measure
Global Appraisal of Individual Needs (GAIN)	13
Teen Addiction Severity Index (T-ASI)	4
Adolescent Diagnostic Interview (ADI)	4
Problem Oriented Screening Instrument for Teenagers (POSIT)	2
Child Adolescent Functional Assessment Scale (CAFAS)	1
Washington Association of Juvenile Court 6Administrators - Risk Assessment (WAJCA-RA)	1
Adolescent Drug Abuse Diagnosis (Euro-ADAD)	1

**Table 6:** Evaluation of multidimensional instruments covering five or more systems

Selection criterion	T-ASI	Euro-ADAD	CAFAS	GAIN	POSIT	WAJCA-RA	ADI
1) feasibility discrete choice experiment: # domains < 10	7	8	8 (+2 optional caregiver domains)	8	10	12	12
2) practicability of assessment in clients: - # items < 500 - tta < 60 min.	154	150	165	1606			
	30-45	45-55	10	60-120			
3) accessibility: price and availability of digital version	Digital version available free of charge	Digital version available free of charge	Available via paid online system (price $78-$400)				
4) psychometric properties [validity (content validity, construct validity, sensitivity to change); reliability (test/retest reliability, internal consistency)]	*Kaminer, 1991:* “…interrater reliability is very good; indeed, it equals or surpasses that found for most interview instruments…” “Although further refinement needs to be made with respect to the Family Relationship ScaIe, the other individual scale ratings and the overall rating agreement is quite high, underscoring the reliability of the T-ASI.” (N=25)	*Czobor, 2011:* Good test-retest reliability (Pearson’s r >= 0.8 for most individual domains) and internal consistency (“Based on Cronbach’s coefficient alpha the internal consistency/reliability of the instrument was 0.50… when we applied an extension of the internal consistency/ reliability measure for multidimensional case, we found that the internal consistency/reliability was satisfactory.”); good criterion, convergent and discriminant construct validity. (N=632)					
	*Kaminer, 1993*: “…1) T-ASI discriminated between psychoactive substance use disorders (PSUD) and non-PSUD in adolescents within a group of psychiatric inpatients; 2) T-ASI substance use, psychiatric status, family function, and school status scores were related to external criteria; 3) there was specificity in these relations with external criteria…support for the valid psychometric properties of the T-ASI….All results should be interpreted in the context of the small sample size (N=25).”						
5) frequency of use in the field: - number of times used in studies	4	1					

Two psychometric studies with small sample sizes were identified for the T-ASI [43, 53] and one study [33] with a larger sample size was identified for the Euro-ADAD. Frequency of use was slightly favorable for the T-ASI compared to the Euro-ADAD as the instrument was used four times in the studies identified in this systematic review, whereas the Euro-ADAD was used in no more than one study. These differences were not considered sufficient to justify favoring either of the instruments over the other. Hence, the T-ASI and Euro-ADAD were considered to have equal potential suitability for the comprehensive evaluation of systemic family interventions in a health economic framework.

## Discussion and conclusions

The objective of this systematic literature review was to identify existing instruments in the field of adolescent delinquency and substance use, which cover the relevant domains of systemic family interventions. The instruments were appraised based on characteristics relevant for use in economic evaluations such as brevity, accessibility, psychometric properties etc. Euro-ADAD and T-ASI showed favorable characteristics in relation to the criteria for a comprehensive outcome measure, covering multiple relevant systems and being suitable for obtaining preference weights. Both instruments lack preference weights for the outcomes, at present. Attaining these (as a potential next step) would facilitate calculating ‘utility scores’ as common in economic evaluations. Furthermore, the results of the current study may inform future efforts towards standardized and comprehensive core outcome sets as defined by the COMET initiative [54]. The study may be seen as a preparatory step towards a full COMET effort to standardizing the QALY approach to include broader effects.

Some limitations of this study must be noted. First, given our focus on published research up to 2013, we may have missed out on very recent developments in this field. In the Netherlands, for instance, a new, comprehensive instrument for measuring substance abuse in adolescents is being developed, called the MATE-Y [55], which includes nine modules each containing several domains. Yet, up to today there have not yet been publications on the MATE in the field of youth/adolescents. But similar developments may be ongoing elsewhere. Second, we have not investigated the possibility of constructing a new measure by combining different measures into one composite measure. Though this may be a limitation of this paper, we considered it a necessary first step to identify the instruments currently available in the field for direct use. This may also help to highlight the relevant domains to include in a newly developed instrument. With our approach, we were able to identify two instruments as most promising candidates to use in comprehensive evaluations of systemic family interventions. Neither instrument is currently considered ‘gold standard’ in practice. Furthermore, as common for systematic reviews, the results from the current study are based on a limited selection of databases within a limited timeframe. Yet the number of screened and identified articles was extensive and we assume that the consultation of an even larger number of databases would not have yielded significant differences in results. Also, the characteristics for further selection of the instruments were necessarily arbitrary and guided by our goal of selecting one or more instruments suitable to be used to attain societal preference weights and be used in economic evaluations in the long term. We realize that the suitability criterion of a maximum of 500 questions/1 h of completion time may be rather high when considering the busy clinical practice and ongoing evaluation of patient progress. Furthermore, had we considered different or more broad characteristics, additional instruments might have been found suitable. For example, one could think of shortening existing longer instruments first and then proceeding towards steps of attaining societal preference weights. In the light of limited time, this was not considered feasible in the current study.

Notwithstanding these limitations, our review revealed two promising, currently used instruments, which may be made suitable for inclusion in economic evaluations of systemic family interventions: the Euro-ADAD and T-ASI. To make these instruments suitable for health economic evaluations, first of all, more detailed investigation is necessary of their validity, feasibility and comprehensiveness. Current information on this is scarce, yet needed. Moreover, using these instruments in health economic evaluations will require important next steps. In particular, preference weights would need to be derived for the different states described by the instrument, like those available for health-related utility measures such as the EQ-5D. This is possible through preference elicitation techniques, such as discrete choice experiments or time-trade-off techniques, ultimately leading to ‘utility scores’, which can be attached to the different ‘states’ described by the instrument.

Intriguing questions in this context relate to who should indicate the state a person is in and who should provide the values for the different possible states (i.e., whose preferences count). In line with many guidelines for health-economic evaluations [11, 56], and in line with the broad aim of systemic family interventions, one could ask ‘patients’ to provide self-reports based on one of the identified multidimensional instruments. The value attached to this state could then be based on preferences obtained in the general population. This would provide ‘societal weights’ for the broad outcomes of systemic family interventions. These societal weights could thus be attached to the state a person indicates him- or herself to be in on the multidimensional instrument, thus leading to an overall utility score. Given the broad range of outcomes, including effects incurred by others than the patient or even his family (e.g., a safe neighborhood), the score thus relates to a preference ordering over states that include the effects on more than the patient alone. This may be an additional reason for opting for general public preferences. However, whether the general public is the appropriate source (rather than e.g. decision makers or health care professionals) must be further assessed and discussed, as well as their ability to appropriately weight such diverse outcomes. The more fundamental question is whether these scores would count as ‘utilities’ or rather as multi-criteria decision weights.

Other relevant issues in developing a multidimensional utility measure of systemic family interventions may be the diversity and hierarchy of treatment effects. As mentioned earlier, a comprehensive measure would include health as well as non-health effects and would also include both the effects on the patient himself and society as a whole. Obviously, these different effects may be interrelated. Moreover, some observable effects may be considered to be intermediate effects, whereas others may be final outcomes. Related to this point, there may be short-term and long-term effects, which can be important. Hence, in the construction of such a preference-based measure, good care needs to be taken of the possible interaction of the effects.

One may argue that an alternative route to finding an appropriate outcome measure could be to use existing measures in the field of economic evaluation, most notably QALY measures. To our knowledge, so far there have been only a few studies on the validity of preference-weighted health-related quality of life instruments in an adult population of substance abusers [57, 58]. There have been two studies on the degree to which common preference-weighted measures of quality of life (e.g. QWB-SA, SF-12) correlate with substance use severity [58, 59]. Whereas the first study provides evidence for insufficient coverage of all disease dimensions in substance use disorder [58], the second study does suggest moderate to good correlation between quality of life measures and substance use severity measures [59]. In order to verify these results and determine whether the proposed instruments add value in the field of delinquency and substance abuse in adolescents, further research on the suitability and potential of the quality adjusted life year (QALY) measure in this population is recommended.

Keeping these alternatives in mind, further research on the instruments highlighted in the current paper, specifically on the attachment of societal preference weights could bring evaluation of mental health interventions for delinquent and substance abusing adolescents closer to the standard methodology in health economic evaluations of curative medical interventions. Both identified instruments appear suitable and broad enough to capture the effects of family interventions in substance abusing and delinquent adolescents in such CUA. Adding societal preference weights to one of these instruments will create an instrument, which combines the advantage of the specificity of a disorder-specific instrument with compliance with common methodology of health economic evaluations and captures the broad effects relevant to mental health interventions. CUAs of these interventions can then be performed based on a broad and specific measure that includes several systems/dimensions and at the same time acknowledges the relative value that society attaches to improvements in these diverse systems. Though performing CUAs in the field of substance abuse and delinquency in adolescents remains a challenging task, this paper attempted to contribute to confronting one of the major issues in that context: finding a suitable outcome measure.

## Abbreviations

AARS: Adolescent Assessment/Referral System
ADAD: Adolescent Drug Abuse Diagnosis
ADI: Adolescent Diagnostic Interview
ASI: Addiction Severity Index
BSFT: Brief Strategic Family Therapy
CAFAS: Child Adolescent Functional Assessment Scale
CBCL: Child Behavior Checklist
CBT: Cognitive Behavioral Therapy
CUA: Cost-utility analysis
EBFT: Ecologically Based Family Therapy
ERIC: Education Resource Information Center
EuroADAD: European Adolescent Assessment Dialogue
FFT: Functional Family Therapy
GAIN: Global Appraisal of Individual Needs
GAIN-SS: Global Appraisal of Individual Needs Short Screener
MDFT: Multidimensional Family Therapy
MET: Motivational Enhancement Therapy
MST: Multisystemic Therapy
POSIT: Problem Oriented Screening Instrument for Teenagers
QALYs: Quality-Adjusted Life-Years
